# Strategies for Zinc Uptake in *Pseudomonas aeruginosa* at the Host–Pathogen Interface

**DOI:** 10.3389/fmicb.2021.741873

**Published:** 2021-09-08

**Authors:** Shuaitao Wang, Juanli Cheng, Yanting Niu, Panxin Li, Xiangqian Zhang, Jinshui Lin

**Affiliations:** ^1^College of Life Sciences, Yan’an University, Yan’an, China; ^2^Shaanxi Key Laboratory of Chinese Jujube, Yan’an University, Yan’an, China

**Keywords:** *Pseudomonas aeruginosa*, zinc uptake system, Zur, zincophore, nutritional immunity

## Abstract

As a structural, catalytic, and signaling component, zinc is necessary for the growth and development of plants, animals, and microorganisms. Zinc is also essential for the growth of pathogenic microorganisms and is involved in their metabolism as well as the regulation of various virulence factors. Additionally, zinc is necessary for infection and colonization of pathogenic microorganisms in the host. Upon infection in healthy organisms, the host sequesters zinc both intracellularly and extracellularly to enhance the immune response and prevent the proliferation and infection of the pathogen. Intracellularly, the host manipulates zinc levels through Zrt/Irt-like protein (ZIP)/ZnT family proteins and various zinc storage proteins. Extracellularly, members of the S100 protein family, such as calgranulin C, sequester zinc to inhibit microbial growth. In the face of these nutritional limitations, bacteria rely on an efficient zinc transport system to maintain zinc supplementation for proliferation and disruption of the host defense system to establish infection. Here, we summarize the strategies for zinc uptake in conditional pathogenic *Pseudomonas aeruginosa*, including known zinc uptake systems (ZnuABC, HmtA, and ZrmABCD) and the zinc uptake regulator (Zur). In addition, other potential zinc uptake pathways were analyzed. This review systematically summarizes the process of zinc uptake by *P. aeruginosa* to provide guidance for the development of new drug targets.

## Introduction

The ions of numerous metals, notably magnesium, calcium, zinc, iron, manganese, and copper, play numerous biological roles as both structural and catalytic cofactors for proteins (Andreini et al., 2008). For example, zinc participates in the structure of more than 2,000 transcription factors and is a cofactor for more than 300 enzymes (Josune et al., 2017), including hydrolases, transferases, oxyreductases, ligases, isomerases, and lyases (Baltaci et al., 2017). It is estimated that the activity of one in three proteins requires metal ions (Gray, 2003; Dupont et al., 2006). These metal ions are also essential to microbial pathogens during infection because they are involved in bacterial metabolism and various virulence factor functions. Therefore, during infection, bacteria need to acquire biological metal ions from the host, which results in competition for these ions (Waldron and Robinson, 2009). To combat invading pathogens, vertebrate hosts exploit the requirement for nutrient metals by limiting their availability, which is a process termed ‘nutritional immunity’ (Kehl-Fie et al., 2011). This restriction starves invaders of these essential metals, thereby inactivating metal-dependent processes, reducing bacterial growth, and rendering them more sensitive to other aspects of the immune response (Grim et al., 2017). Nutritional immunity is the result of host defense strategies against microbial invaders based on deprivation of, or poisoning with, metals. Although originally associated only with iron restriction, it is now known that other metals, including zinc and manganese, are also sequestered during infection (Juttukonda and Skaar, 2017).

*Pseudomonas aeruginosa* is an ubiquitous Gram-negative bacterium that causes nosocomial infections, as well as fatal infections in immunocompromised individuals (Wu et al., 2015). This organism is one of the top three causes of opportunistic human infections, and a major factor in its prominence as a pathogen is its intrinsic resistance to antibiotics and disinfectants (Lin and Cheng, 2019). Indeed, *P. aeruginosa* accounts for 10–20% of nosocomial infections (Fazeli et al., 2012) and can cause hospital-acquired pneumonia (HAP) along with ventilator-associated pneumonia (Gales et al., 2001), gastrointestinal infections, dermatitis, urinary tract infections (UTIs), skin infections, bacteremia, soft tissue infections (Azam and Khan, 2019), respiratory system infections in patients with cystic fibrosis (CF; Gales et al., 2001), bone and joint infections, and several other infections in patients with severe burns and immunocompromised patients, such as those with cancer or acquired immune deficiency syndrome (AIDS; Tacconelli, 2002; Azam and Khan, 2019). In 2017, *P. aeruginosa* was recognized as one of the most life-threatening bacteria and listed as a priority pathogen for research and development of new antibiotics by the World Health Organization (Fadi et al., 2018; Azam and Khan, 2019).

It is clear that metals play key roles during infection and in the battle between pathogens and hosts (Weinberg, 2009). Zinc is an essential nutrient required at low concentrations by almost all living organisms (Schalk and Cunrath, 2016). This trace element is the second most abundant in organisms after iron (Schalk and Cunrath, 2016; Gonzalez et al., 2018). In the opportunistic bacterial pathogen *P. aeruginosa*, zinc has been shown to play important roles in virulence, colonization of the host organism, and antibiotic resistance (Gonzalez et al., 2018). *P. aeruginosa* has evolved a variety of zinc transport systems that enable it to thrive in zinc-deficient environments and during infection (Gonzalez et al., 2018). Here, we summarize current knowledge about strategies that use zinc for host–pathogen interactions, along with host strategies to limit pathogenic bacterial zinc absorption. We focus on the introduction of three known zinc uptake systems (ZnuABC, HmtA, and ZrmABCD) and zinc uptake regulator (Zur) in *P. aeruginosa* while discussing other potential zinc uptake systems. The purpose of this review is to provide guidance for the development of new drugs that utilize the zinc uptake system of *P. aeruginosa*.

## The Contribution of Zinc to the Host–Pathogen Interaction

### Biochemical Properties of Zinc

Zinc was first shown to be required for the growth of *Aspergillus niger* by Raulin (1869). Since then, zinc has been demonstrated to be essential for the growth, development, and differentiation of all life forms, including microorganisms, plants, and animals (Vallee and Auld, 1992). Because zinc is characterized by complete d orbitals, it does not participate in redox reactions, but instead functions as a Lewis acid to accept pairs of electrons (Mccall et al., 2000). Therefore, Zn^2+^ is an ideal metal cofactor for reactions that require a redox-stable ion to function as a Lewis acid-type catalyst, such as proteolysis and the hydration of carbon dioxide (Butler, 1998). Because of these properties, Zn^2+^ is involved in a broad range of biological functions, including structural roles through stabilization of secondary, tertiary, or quaternary protein conformations, as well as catalytic roles because of its electrophilic properties (Gonzalez et al., 2018). Zn^2+^ is also an essential antioxidant mineral for prevention of the formation and reaction of free radicals. Intracellular free zinc ions can inhibit the Fenton reaction by competing with ferrous ions to reduce the toxic effects of reactive oxygen radicals toward cells (Faulkner and Helmann, 2011). In cells, zinc is typically buffered and bound to metalloproteins (5–6% of the proteome in prokaryotes, 9–10% of the proteome in eukaryotes), but it may also exist in a labile or chelatable (free ion) form (Andreini et al., 2006). Through molecular interactions with biomolecules, zinc participates in a wide variety of metabolic processes as well as in the repair and maintenance of cell structures and biomolecules, including transcriptional regulation (Mikhaylina et al., 2018), RNA and DNA synthesis (Andreini et al., 2006), DNA replication (Nejdl et al., 2014), cell growth, apoptosis (Andreini et al., 2006), and the endocrine and immune systems (Wa̧tły et al., 2016; Gonzalez et al., 2018).

### Zinc Deficiency Increases Host Sensitivity to Pathogens

Zinc is essential to host immune function, and even mild zinc insufficiency leads to widespread defects in both innate and adaptive immunity, resulting in impaired clearance of pathogens, augmented initial inflammatory response, and collateral damage to host tissues (Kuźmicka et al., 2020; Wang et al., 2020). The innate immune system is a diverse collection of cells and proteins made up of monocytes, macrophages, and neutrophils that can trigger inflammation and antibacterial responses and prevent alien invasions (Wang et al., 2020). At sites of infection, epithelial cells form the first and highly effective barrier layer, but if they are breached, a rapid influx of phagocytes such as neutrophils and macrophages will assist in curbing the initial progress of infection. Engulfment of pathogens by these phagocytes helps activate adaptive immunity, which leads to a permanent resolution of the infection (Djoko et al., 2015). In innate immunity, zinc deficiency not only diminishes the recruitment, migration, and differentiation of monocytes (Kojima et al., 2007; Baltaci et al., 2017), but also reduces the chemotactic response of macrophages and neutrophils, resulting in impaired phagocytosis and intracellular killing of pathogens (James et al., 1987; Wirth et al., 1989; Djoko et al., 2015). In adaptive immunity, zinc deficiency causes a decrease in the percentage of T cells and lytic activity of NK cells (Maares and Haase, 2016; Baltaci et al., 2017). Nutritional zinc deprivation also causes a significant loss of premature and immature B cells and decreases antibody production (Lin et al., 2018; Cwa et al., 2020; von Pein et al., 2021). Thus, numerous animal and human studies indicate that zinc deficiency decreases resistance to infectious diseases. Zinc-deficient animals have suppressed immune responses and are more susceptible to a diverse range of infectious agents, including Herpes simplex virus (Feiler et al., 1982) and Semliki forest virus (Singh et al., 1992), bacteria such as *Francisella tularensis* (Pekarek et al., 1977), *Listeria monocytogenes* (Carlomagno et al., 1986; Coghlan et al., 1988), *Salmonella enteritidis* (Kidd et al., 1994), and *Mycobacterium tuberculosis* (McMurray et al., 1990), the protozoan parasites *Trypanosoma cruzi* (Fraker et al., 1982), *Trypanosoma musculi* (Lee et al., 1983), *Toxoplasma gondii* (Tasçi et al., 1995), and *Plasmodium yoelii* (Shankar et al., 1995), eukaryotes such as *Candida albicans* (Salvin et al., 1987; Singh et al., 1992), and the helminths *Heligmosomoides polygyrus* (Minkus et al., 1992; Shi et al., 2010), *Strongyloides ratti* (Fenwick et al., 1990), *Trichinella spiralis* (Fenwick et al., 1990), *Fasciola hepatica* (Flagstad et al., 1972), and *Schistosoma mansoni* (Nawar et al., 1992). In summary, zinc deficiency affects cells involved in innate and adaptive immunity at the level of survival, proliferation, and maturation and increases host sensitivity to bacterial, viral, and fungal infections.

### Zinc Deficiency Reduces Pathogen Infection Ability

Immediately after invasion by infecting bacteria, the body will initiate a nutritional immune response, reducing free zinc levels in the blood and tissue (Graham et al., 2009; Juttukonda and Skaar, 2017). By doing so, the host can not only prevent zinc acquisition by bacterial pathogens, thereby limiting bacterial growth and proliferation, but also redistribute zinc to different cells, thereby enhancing immune function (Kehl-Fie and Skaar, 2010; Ying et al., 2015). Although bacteria are characterized by direct contact with their surrounding environment, they can thrive under metal limitation conditions through several metal transport mechanisms. Therefore, the main consideration with regard to zinc at the host–pathogen interface has been the role of nutritional immunity (Kehl-Fie and Skaar, 2010; Ying et al., 2015). The competition for zinc has recently been considered a possible target for new antimicrobial therapies (Cuajungco et al., 2021). Bacterial competition for zinc ions (Zn^2+^) exists as Zn^2+^ is used by bacteria to maintain cell structure and physiological functions. It also affects the interplay between bacterial virulence factors and pathogenic processes (Xia et al., 2021). For instance, most bacterial pathogens use zinc-dependent microbial metalloproteinases to facilitate the destruction of physiological barriers during host invasion by pathogens, thereby assisting in the pathogenesis (Rahman and Karim, 2017). Some research shows zinc is capable of forming complexes with certain antibiotics, such as penicillin and tetracyclines, leading to their degradation or inactivation (Eisner and Porzecanski, 1946; Novák-Pékli et al., 1996). Zinc also mediates protection against the adverse effects of reactive oxygen species (ROS) that are produced during inflammatory processes (Rahman and Karim, 2017). As a result, the resistance of bacteria to antimicrobial substances and host immune response are improved (Rahman and Karim, 2017). Furthermore, studies have shown that zinc promotes biofilm formation in *P. aeruginosa* and *Xylella fastidiosa*, which leads to difficulty in eradicating pathogens from the host (Kumar et al., 2017; Kandari et al., 2021). Interestingly, Zn^2+^ can also regulate transcription of RNA *via* regulation of the activity of transcription factors and several enzymes, such as RNA and DNA polymerases (Nejdl et al., 2014). Two-component signal transduction systems (TCS) are the most important mechanisms used by bacteria to detect and respond to changing environmental conditions and stresses (Stock et al., 2000). In situations of Zn^2+^ excess, the CzcRS two-component system of *P. aeruginosa* will be activated by Zn^2+^ and induce an export mechanism (Chambonnier et al., 2016). This not only improves the bacteria’s ability to resist metals, but also facilitates survival during interactions with other living organisms, particularly after phagocytosis by eukaryotic cells (Djoko et al., 2015; Chambonnier et al., 2016). In short, zinc plays many essential roles within bacterial pathogens. In addition to acting as a necessary cofactor for cellular proteins, making it indispensable for both protein structure and function, they also fulfill roles in signaling and regulation of virulence.

## The Host Uses Nutritional Immunity to Limit the Acquisition of Zinc by Pathogenic Bacteria

Mammalian zinc homeostasis is highly complex and can involve tissue-specific expression of specialized Zn^2+^ transporters, as well as metal transcription factors (MTF-1/2) and zinc storage proteins (Djoko et al., 2015). There are two families of mammalian zinc transporters exist. The first is the ZnT family of transporters, which consists of 10 genes (*SLC30A1* to *SLC30A10*) that decrease intracellular zinc levels by transporting zinc from the cytoplasm to the lumen of organelles or the extracellular space. The second is the Zrt/Irt-like protein (ZIP) family, which consists of 14 genes (solute carrier family 39 from *SLC39A1* to *SLC39A14*). Proteins in the ZIP family increase intracellular zinc levels by transporting the metal from either the extracellular space or organellar lumen into the cytoplasm (Lichten and Cousins, 2009). The metal-response-element-binding transcription factor (MTF)-1 is able to sense free Zn^2+^ through its six zinc-finger domains, resulting in stabilization of the level of Zn^2+^ through the feedback-regulation of metallothionein (MT) and Zn^2+^ transporter transcriptions (Lichten et al., 2011). Cytosolic free Zn^2+^ is sequestrated into ubiquitously expressed MT and intracellular organelles, such as the endoplasmic reticulum (ER), Golgi, lysosomes, and even high amounts of zinc-containing membrane-bound vesicular structures known as zincosomes (Rahman and Karim, 2017; Wang et al., 2020).

**FIGURE 1:**
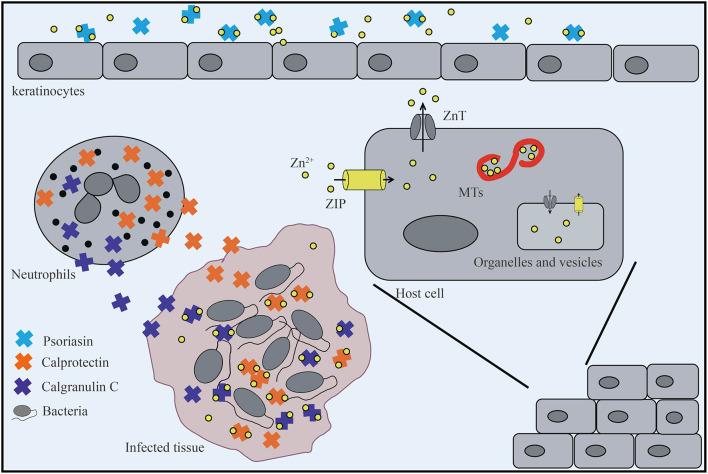
The host limits the absorption of zinc by bacteria. Keratinocytes secrete psoriasin to sequester metals and prevent infection. In the infected tissue, neutrophils that release calprotectin and calgranulin C are recruited, which inhibits bacterial invasion by chelating Zn^2+^. In addition, host cells mobilize Zn^2+^ into or out of the cytoplasm through two tissue-specific Zn^2+^ transporters: Zrt/Irt-like protein (ZIP) and ZnT. In the cytosol, metallothioneins (MTs) bind Zn^2+^ to reserve, buffer, and chelate and enter or leave intracellular organelles and vesicles through ZnT and ZIP transporters.

Upon infection in healthy organisms, host cells use members of the ZIP transporter family to introduce Zn^2+^ into the cells to prevent the proliferation and infection of pathogens. Zn^2+^ is then distributed to the intracellular organelles or binds to MT through ZnT family proteins to limit the concentration of intracellular Zn^2+^ (Figure 1; Djoko et al., 2015; Xia et al., 2021). During this process, zinc is redistributed to various tissues such as the liver, resulting in decreased serum zinc levels. This process can occur through the IL-6-dependent upregulation of ZIP14, which is the zinc transporter responsible for accumulating zinc in hepatocytes (Liuzzi et al., 2005). In this process, plasma zinc is also depleted, and the mechanism appears to involve MT, which accumulates in the liver in its zinc-bound form following stimulation by inflammatory cytokines such as IL-1 (Cousins and Leinart, 1988).

In the process of bacterial infection, the host not only uses specifically expressed ZIP/ZnT transporters and zinc storage proteins to transfer and store bacterial-accessible zinc, but also utilizes nutritional immunity to further isolate metal ions from pathogens to reduce their growth and control infection. Numerous eukaryotic zinc-binding proteins possess both Zn^2+^ chelating and pro-inflammatory properties and therefore assist in host-mediated antimicrobial activity. Examples of these proteins include psoriasin, calprotectin, and calgranulin C (Figure 1; Moroz et al., 2003; Gläser et al., 2005), which belong to the S100 protein family of Ca^2+^ binding proteins in vertebrates (Hood and Skaar, 2012). Psoriasin (also known as S100A7) is secreted by keratinocytes and acts as an effective chemical barrier in epithelial cells *via* zinc chelation to inhibit microbial growth (Hattori et al., 2014). Calprotectin (also known as S100A8/S100A9, calgranulin A/B, MRP8/14) is a heterodimer of S100A8 and S100A9 and a bivalent metal-binding protein released by neutrophils (Gebhardt et al., 2006) that was originally identified based on its ability to inhibit the growth of a variety of fungal and bacterial pathogens *in vitro* (Sohnle et al., 1991; Cassat and Skaar, 2012). Upon pathogen attack, calprotectin, which is one of the most abundant antibacterial proteins in neutrophils, is recruited to chelate zinc ions at the site of infection (Xia et al., 2021). This chelation is mediated through two high-affinity binding sites, both of which can bind Zn^2+^ with nanomolar affinity (Kehl-Fie et al., 2011). Calprotectin is thought to induce zinc limitation as a means to control infections caused by *Staphylococcus aureus* and *Acinetobacter baumannii* in tissues, as well as *Clostridium difficile* and *Salmonella enterica* serovar Typhimurium in the gastrointestinal tract (Zackular et al., 2016; Vermilyea et al., 2021). For example, studies have shown that, in mice colonized with *C. difficile*, excess dietary zinc intensifies the severity of *C. difficile*–associated disease, and calprotectin is an essential host factor for combating *C. difficile* by limiting zinc availability during infection (Zackular et al., 2016). Moreover, recent studies have demonstrated the potential for calprotectin-mediated zinc chelation to post-translationally inhibit zinc metalloprotease activity and thereby impact the protease-dependent physiology and/or virulence of *P. aeruginosa* in the CF lung environment (Vermilyea et al., 2021).

In addition to calprotectin, neutrophils also express calgranulin C (S100A12), which binds both zinc and copper *in vitro* and possesses antimicrobial activity (Carvalho et al., 2020). The zinc-binding protein calgranulin C primarily participates in superoxide formation to exert its antibacterial activity, although it also affects ubiquitin and beta-catenin degradation in host cells by interacting with S100A9 or directly with calcyclin-binding protein and Siah-1-interacting protein (SIP; Filipek et al., 2002; Carvalho et al., 2020). In addition to their metal-chelating properties, psoriasin, calprotectin, and calgranulin C can bind to and activate cell surface receptors, such as Toll-like receptor 4 (TLR4), receptor for advanced glycation end-products (RAGE), and G protein-coupled receptors (GPCRs) to initiate intracellular inflammatory signal transduction. These proteins also play an important role in the regulation of immune and inflammatory responses (Carvalho et al., 2020; Xia et al., 2021).

In short, the host prevents the proliferation and infection of pathogens by enhancing its immune response to pathogens. Intracellularly, the host manipulates zinc levels through ZIP/ZnT family proteins and various zinc storage proteins. Extracellularly, members of the S100 protein family, such as calgranulin C, sequester zinc to inhibit microbial growth. In the face of these nutritional limitations, bacteria rely on an efficient zinc transport system to maintain zinc supplementation for proliferation and disruption of the host defense system to establish infection (Figure 1; Schalk and Cunrath, 2016; Gonzalez et al., 2018; Cuajungco et al., 2021; Kandari et al., 2021).

## Zinc Uptake Systems Mediate the Adaptation of *P. aeruginosa* to Zinc-Deficient Environments

**FIGURE 2:**
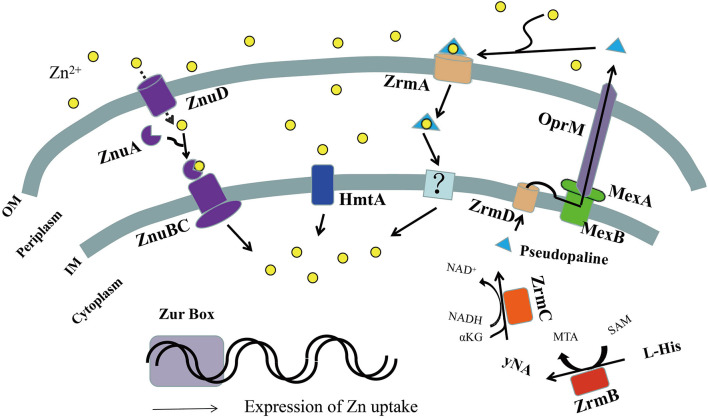
Schematic representation of zinc uptake systems in *Pseudomonas aeruginosa*. In zinc-limited environments, the transcription regulator zinc uptake regulator (Zur) could not bind DNA as a repressor, leading to the expression of zinc uptake systems. The TonB-dependent transporters (ZnuD) located in the outer membrane allow the import of extracellular Zn^2+^ in the free form, directly into the periplasm. The periplasmic space protein ZnuA transmits it to the ZnuBC transporter complex of the inner membrane, further inside the cytoplasm. Periplasmic Zn^2+^ can also be imported into the cytoplasm *via* the P-type ATPase, HmtA. Pseudopaline synthesized by ZrmBC is released into the periplasmic space through the plasma membrane transporter ZrmD, then exported into the extracellular space through the MexAB-OprM efflux pump, where free Zn^2+^ ions are chelated extracellularly to form the pseudopaline-Zn^2+^ complex. The complex is then transported into the periplasmic space by the outer membrane receptor ZrmA and moved into the cell by an unknown inner membrane transporter or unloaded Zn^2+^ to complete extracellular Zn^2+^ uptake. OM, outer membrane; IM, inner membrane.

Many diseases are caused by bacterial infections, such as bacteremia, UTIs, respiratory system infections, and burn infections. *P. aeruginosa* is usually the main pathogenic strain in these infections (Fazeli et al., 2012; Ding et al., 2016; Streeter and Katouli, 2016). Because of its strong resistance to a variety of antibiotics, *P. aeruginosa* brings great challenges to clinical treatment. During infection, *P. aeruginosa* activates the nutritional immune response in the host, resulting in the chelation of essential elements such as zinc by a variety of host proteins (Azam and Khan, 2019; Kandari et al., 2021; Vermilyea et al., 2021). Therefore, to ensure successful infection, *P. aeruginosa* must adapt to zinc-deficient environments, and this process is mainly mediated by zinc uptake systems (Maares and Haase, 2016; Schalk and Cunrath, 2016; Gonzalez et al., 2018; Kandari et al., 2021). At present, there are three known zinc uptake systems in *P. aeruginosa*, ZnuBC, HmtA, and ZrmABCD (Figure 2), which are all regulated by the Zur protein (Lewinson et al., 2009; Ellison et al., 2013).

Deletion of *znuABC* from *P. aeruginosa* reduced intracellular zinc ion accumulation by 60%, which lessened alginate production, reduced the activity of extracellular zinc-containing proteases, including LasA, LasB, and protease IV, and decreased the ability of *P. aeruginosa* to disseminate during systemic infections (D’Orazio et al., 2015). Deletion of the *zrmABCD* system also significantly reduced the survival rate of *P. aeruginosa* infecting larvae of *Galleria mellonella* (greater wax moth) (Lin et al., 2020) and its virulence and infection ability in mice (Mastropasqua et al., 2017). In addition, the *zur* deletion mutation reduced the production of phenazine and the *Pseudomonas* quinolone signal (PQS; Streeter and Katouli, 2016) and significantly reduced the pathogenicity in neutropenic mice and nematode infection models (Ellison et al., 2013). These results indicate that *P. aeruginosa* uses the zinc uptake system to compete with its host for Zn^2+^ to meet its own nutritional needs and to influence the production of virulence factors involved in colonization.

### ZnuABC: A Specific High-Affinity Zinc Uptake System

A specific high-affinity zinc uptake system was first identified in *Escherichia coli*; namely, the zinc uptake system mediated by ATP binding cassette (ABC) transporter ZnuABC (Luo and Liu, 2019). Bioinformatics analyses of the *P. aeruginosa* PAO1 genome identified three genes homologous to *E. coli znuABC*, PA5498 (*znuA*), PA5500 (*znuB*), and PA5501 (*znuC*). ZnuA is a zinc-specific solute-binding protein (SBP) with high affinity that exists in the periplasmic space (Luo and Liu, 2019). *P. aeruginosa* ZnuA has two Zn^2+^ binding sites, one of which is a high affinity site composed of three histidine residues, His60, His140, and His204 that has a dissociation constant *K*_*d*_ to Zn^2+^ of 22.6 ± 6.4 nmol/L. The other binding site is a low affinity site with a micro-molar level (Pederick et al., 2015). The ZnuBC transporter consists of ZnuB (an intimal permeable enzyme) and ZnuC (an ATP enzyme) in the inner membrane (Luo and Liu, 2019).

In *Neisseria meningitidis*, ZnuABC is related to the TonB-dependent outer-membrane transporter ZnuD, which is responsible for transporting extracellular Zn^2+^ across the outer-membrane (Calmettes et al., 2015). *P. aeruginosa* also encodes a protein PA0781, which shares 27% identity with ZnuD. *znuA* deletion mutation significantly induces PA0781 expression under Zn^2+^-restricted conditions, which suggests that PA0781 may be a homologous protein of ZnuD (Pederick et al., 2015).

In summary, the ZnuD homologous protein (PA0781) facilitates Zn^2+^ recruitment to the periplasm in *P. aeruginosa*, and ZnuA (the periplasmic space protein) transmits it to the ZnuBC transporter complex of the inner membrane, and further inside the cytoplasm, thereby enabling subsequent import of Zn^2+^ to the cytoplasm (Figure 2). Although ZnuABC plays a role in the zinc uptake of *P. aeruginosa*, absence of the putative *znuA*, *znuB*, or *znuC* genes only slightly reduces its growth in zinc depleted conditions relative to the wild-type strain PAO1 (Ellison et al., 2013; Pederick et al., 2015), indicating that there are other zinc uptake systems in *P. aeruginosa*.

### HmtA: A P-Type ATP Inner-Membrane Transporter

In addition to the ZnuABC, HmtA (PA2435) is also one of the zinc uptake systems in *P. aeruginosa*. HmtA is a P-type ATPase located in the inner membrane of *P. aeruginosa* (Lewinson et al., 2009). P-type ATPases constitute a superfamily of transporters characterized by the ability to hydrolyze ATP. The hallmark of this family of pumps is the formation of a phosphoenzyme intermediate (hence the name P-type ATPase) by transfer of the γ-phosphate from ATP to the highly conserved DKTGT motif (Dao et al., 2016). A family of P-type ATPases catalyzing the translocation of transition metals (also referred to as heavy-metal or type P_1__*B*_ ATPases) has been identified. Such P-type ATPases harbor a Cys-Pro-Xaa (or Xaa-Pro-Cys) motif, with Xaa being Cys, Ser, or His in their sixth transmembrane helix (TM6) that is essential for transport activity (Argüello, 2003). Different P-type ATP transporters have different substrate specificities, but most are responsible for the efflux of Ag^+^/Cu^+^ or Zn^2+^/Cd^2+^/Pb^2+^ (Lewinson et al., 2009). However, the difference of most P-type ATP enzymes used as efflux pumps is that HmtA is a metal ion input protein, which is highly selective to the substrate. HmtA mediates the uptake of Cu^2+^ and Zn^2+^ by *P. aeruginosa*, but does not mediate the uptake of other metal ions such as Ag^+^ and Cd^2+^ (Lewinson et al., 2009).

### ZrmABCD: A Metallophore-Mediated Zinc Uptake System

Under conditions where metal is scarce, a common bacterial strategy in the biosynthesis of metallophores is their export to the extracellular medium and recovery of a metal-metallophore complex through dedicated membrane transporters. Therefore, it has been suggested that, similar to iron chelator-mediated cell iron uptake process, *P. aeruginosa* may also release substances that chelate zinc compounds or proteins (e.g., zinc chelators, or zincophores) to mediate zinc uptake (Hood and Skaar, 2012). Analysis of the genome-wide transcriptional responses of *P. aeruginosa* PA14 to zinc restriction revealed that this type of zinc uptake system, PA4837-PA4834 (named ZrmABCD or CntOLMI), which is regulated by the Zur, is involved in the biosynthesis and trafficking of a staphylopine-like metallophore named pseudopaline (Lhospice et al., 2017; Mastropasqua et al., 2017; Lin et al., 2020; Gomez et al., 2021). The first gene of this operon encodes a TonB-dependent outer membrane protein (ZrmA) that has high homology with siderophore uptake systems (Mastropasqua et al., 2017). This protein mediates transport of the extracellular pseudopaline-Zn^2+^ complex to the intracellular space (Lhospice et al., 2017). Inactivation of the ZrmA gene markedly decreases the ability of *P. aeruginosa* to grow in zinc-limited media and compromises intracellular zinc accumulation (Mastropasqua et al., 2017).

ZrmB and ZrmC, which are responsible for the synthesis of Pseudopaline (Lhospice et al., 2017) have chemical structures and biosynthesis pathways similar to those of the *S. aureus* metallophore staphylopine (Ghssein et al., 2016). Staphylopine is a metallophore distantly related to plant nicotianamine that contributes to the broad-spectrum abilities of *S. aureus* to take up metals including zinc, iron, nickel, cobalt, and copper (Ghssein et al., 2016). Pseudopaline differs from staphylopine with regard to the stereochemistry of its histidine moiety associated with an alpha ketoglutarate moiety instead of pyruvate (Lhospice et al., 2017). The biosynthesis pathway of pseudopaline is composed of two steps: First, under the catalysis of ZrmB, S-adenosyl methionine (SAM) engages the α-aminobutyric acid group on L-histidine (L-His) through nucleophilic attack to produce a reaction intermediate (named yNA). Second, under the catalysis of ZrmC, NADH provides reduction power, and the yNA intermediate is condensed with a molecule of α-ketoglutaric acid (αKG) to produce pseudopaline (Lhospice et al., 2017).

The pseudopaline synthesized in the cell is released into the periplasmic space through the plasma membrane transporter ZrmD (Lhospice et al., 2017). Finally, the MexAB-OprM efflux pump secretes pseudopaline across the membrane to the outside of the cell (Gomez et al., 2021).

Our group also verified the function of the *zrmABCD* operon in *P. aeruginosa* PAO1, and our findings were consistent with those reported by Mastropasqua et al. (2017) and Lhospice et al. (2017). The growth curves of ZnuBC, ZrmB, ZrmA, and ZrmD in zinc-rich and zinc-limited environments showed that ZnuBC, ZrmB, and ZrmD participated in *P. aeruginosa* Zn^2+^ uptake. However, the growth of PAO1 in zinc-limited environments was only significantly inhibited when both *znuBC* and *zrmABCD* were absent. Since ZnuBC has been reported as a zinc uptake system, ZrmABCD is a novel zinc uptake system that is functionally complementary to ZnuBC (Lin et al., 2020). Growth curve analysis following the exogenous addition of cell-culture extracts and the 4-(2-pytidylazo) resorcinol (PAR) assay showed that ZrmB in the *zrmABCD* operon participated in Zn^2+^ uptake by controlling the synthesis of pseudopaline. ZrmD acts as a plasma membrane transporter to mediate the secretion of pseudopaline, while ZrmA acts as an outer membrane receptor to mediate the transport of extracellular pseudopaline-Zn^2+^ complex into the cell (Lin et al., 2020).

Based on these results, we propose the following functional model of the *zrmABCD* operon in *P. aeruginosa*. In a zinc-limited environment, pseudopaline synthesized by ZrmBC is released into the periplasmic space through the plasma membrane transporter ZrmD and then exported into the extracellular space through the MexAB-OprM efflux pump, where free Zn^2+^ is chelated extracellularly to form the Pseudopaline-Zn^2+^ complex. The complex is then transported into the periplasmic space by the outer membrane receptor ZrmA and moved into the cell by an unknown inner membrane transporter or unloads Zn^2+^ to complete extracellular Zn^2+^ uptake (Figure 2; Lhospice et al., 2017; Mastropasqua et al., 2017; Lin et al., 2020; Gomez et al., 2021).

### Zinc Uptake Regulator

Zinc uptake regulator is a zinc-sensing transcriptional regulator that belongs to the Fur superfamily of metal-sensing transcriptional regulators (Kandari et al., 2021). This regulator functions as either a repressor or an activator (Mikhaylina et al., 2018) and regulates the expression of a large number of genes in bacteria, including those responsible for zinc import and zinc export, as well as chaperone proteins, metallochaperones, enzymes, virulence factors, and some ribosomal proteins (Fillat, 2014). In the presence of sufficient zinc in the cell, Zur molecules bind and undergo a conformational change that augments zinc DNA binding ability, keeping the genes involved in zinc uptake and mobilization in a repressed state. However, Zur-mediated repression is stopped when the cell encounters a zinc-depleted condition, thereby initiating zinc uptake and mobilization (Choudhry et al., 2020). Zur forms a homodimer in the cytoplasm, and each monomer consists of an N-terminal DNA-binding (DB) domain, a C-terminal dimerization (D) domain, and a hinge loop between the two (Kandari et al., 2021). Zur has at least two zinc binding sites, one highly conserved zinc binding site (C-site) to maintain structural stability, and one or two conserved zinc binding sites (M-site and/or D-site) to regulate DNA binding (Liu et al., 2021). It was previously believed that Fur family proteins employ an open-to-closed mechanism for conformational activation (Liu et al., 2021). However, Liu et al. (2021) studied the XcZur of *Xanthomonas campestris* pv. *Campestris* and found that zinc perception in the regulator site of XcZur induces a closed-to-open conformational change to activate the transcriptional regulator. Later studies showed that DNA binding of XcZur is likely to be a process of induced fit, which could explain why XcZur is able to bind different target DNAs with diverse modes (Liu et al., 2021).

In *P. aeruginosa*, the coding gene *zur* (PA5499, also known as np20) of Zur proteins forms a polycistronic operon with *znuC* and *znuB* (Ellison et al., 2013). This gene has two conserved zinc binding sites. C_118_XXC_121_-X_136_-C_158_XXC_161_ includes two CXXC motifs, in which the four Cys residues are the highly conserved zinc binding sites (C-site) in *P. aeruginosa* Zur. The Zn-finger structure of Cys4 is completely conserved in Zur proteins of all strains and highly conserved in the Fur protein family (Shin et al., 2011). Another highly conserved zinc binding site, the M-site, is composed of two His residues in the H_109_SH_111_ motif and a highly conserved C_104_. This site is zinc-sensitive. When there is excess Zn^2+^, Zur binds additional Zn^2+^ to the M-site (Shin et al., 2011). In *P. aeruginosa*, the DNA binding region at the C-terminal of the Zur protein binds a 17 bp sequence that contains a palindrome sequence centered on a non-conservative nucleotide (Pederick et al., 2015). Our group also confirmed this point and identified the binding site of Zur protein on the *zrmABCD* operon promoter as GCGTTATAGTATATCAT by electrophoretic mobility shift assays (EMSAs; Lin et al., 2020).

### Other Potential Zinc Uptake Systems

Recently, transcriptome analyses of *znuA* deletion mutant in *P. aeruginosa* showed that the identification of three putative transport systems, in addition to *znuABC*, *hmtA*, and *zrmABCD*, significantly upregulated gene expression under Zn^2+^-restricted conditions: PA1922–PA1925, PA2911–PA2914, and PA4063–PA4066 (Pederick et al., 2015; Gonzalez et al., 2018). The presence of a binding site for the transcriptional Zn^2+^ sensor Zur in the promoter regions of these transporter clusters is consistent with the observed transcriptional response to Zn^2+^ depletion and strongly suggests that these pathways are involved in Zn^2+^ acquisition (Schalk and Cunrath, 2016).

In PA1922–PA1925, PA1922 is a TonB-dependent outer membrane receptor, while PA1923 encodes a putative cobaltochelatase involved in cobalamin biosynthesis. PA1924 encodes a putative ExbD homologous protein. TonB-dependent outer membrane receptors rely on ExbD to energize transport, and PA1925 is an unknown protein. Accordingly, PA1922 and PA1924 may form TonB-dependent outer membrane transporters and participate in the transport of free zinc ions from the extracellular to cytoplasmic space (Gonzalez et al., 2018).

The chelated form of Zn^2+^ in *P. aeruginosa* imported into the periplasm may be internalized *via* PA2912–PA2914 (Pederick et al., 2015). Pederick et al. (2015) predicted that PA2911 is also a TonB-dependent outer membrane receptor, PA2912–2914 encodes an ABC transporter, PA2912 is ATPase, PA2913 encodes a periplasmic space binding protein, and PA2914 is an intimal permeable enzyme. The organizational structure of the PA2911/PA2912–2914 transporter is similar to that of the ZnuD/ZnuABC transporter. PA2911 may function in concert with PA2912–PA2914. First, chelated Zn^2+^ is transported from the extracellular to periplasmic space through the outer membrane receptor PA2911. Next, Zn^2+^ binds to the periplasmic space binding protein PA2913 and is transported through PA2913 to the inner membrane transporter complex PA2912/PA2914, which then passes through the inner membrane into the cytoplasm (Pederick et al., 2015; Gonzalez et al., 2018).

The PA4063–PA4066 operon is necessary for the growth of *P. aeruginosa* in the sputum of patients with CF (Gi et al., 2015). This operon also encodes an ABC transporter containing two periplasmic SBPs of unknown function, PA4063 and PA4066. PA4064 and PA4065 are homologous to the antimicrobial peptide resistance transporters SalX and SalY of *Streptococcus salivarius*, respectively (Gi et al., 2015; Turner et al., 2015). PA4063 and PA4066 proteins consist of 196 and 172 amino acid residues, respectively, and SignalP signal peptide sequence analysis showed that the signal peptide cleavage sites of these proteins were located between amino acid residues 17 and 18 and amino acid residues 23 and 24.^1^ In other words, their mature form has only 179 and 149 amino acid residues, respectively. Such a small protein may not be large enough to interact stably with both ligand and ABC transporter transmembrane domains (Pederick et al., 2015). The PA4063 protein has a large number of histidine residues, which is needed to chelate zinc, and may therefore have zinc chelating activity; however, monomeric PA4066 has an insufficient number of histidine residues to coordinate Zn^2+^ (Pederick et al., 2015). PA4063 protein may periplasmically bind to zinc with the assistance of other proteins. The PA4066 protein of *P. aeruginosa* may play other roles in taking up Zn^2+^. Therefore, we speculate that PA4063 binds Zn^2+^ in the periplasmic space with the assistance of a protein and delivers zinc to the PA4064/PA4065 transporter complex located in the intima and further completes the transmembrane transport of Zn^2+^ to the cytoplasm, in which PA4063 acts as a zinc chelating protein in the periplasmic space. PA4064 serves as ATPase to provide energy for the whole operon to mediate Zn^2+^ uptake, while the function of PA4066 protein in the Zn^2+^ uptake process of *P. aeruginosa* is unknown.

In summary, *P. aeruginosa* needs to use a large number of zinc uptake systems to maintain its growth to adapt to the extreme lack of Zn^2+^. Under the condition of zinc limitation, free Zn^2+^ may complete transmembrane transport from the extracellular to cytoplasmic space through zinc uptake systems such as ZnuD/ZnuABC, HmtA, PA1922–PA1925, and PA4063–PA4066. Zinc in chelated form may be absorbed by two zinc uptake systems, ZrmABCD or PA2911/PA2912–PA2914.

## Other Zinc Uptake Pathways of *P. aeruginosa*

### Non-zinc Proteins Replace Their Zinc-Binding Isoproteins to Adapt to Zinc-Depleted Environments

The transcriptome analysis of *znuA* deletion mutants in *P. aeruginosa* under zinc-restricted culture also showed that the expression of *rpmE2* (PA3600), *rpmJ2* (PA3601), and *dksA2* (PA5536) was significantly upregulated (Pederick et al., 2015; Gonzalez et al., 2018). RpmE2 and RpmJ2 are homologous proteins of the 50S ribosomal proteins RpmE (PA5049) and RpmJ (PA4242) in *P. aeruginosa* (Pederick et al., 2015). There are usually two forms of prokaryotic ribosomal proteins, the C^+^ subtype that binds metal ions such as Zn^2+^, and the C^–^ subtype that does not interact with metal ions due to the lack of metal binding residues. This is because the C^–^ subtype ribosomal protein replaces the Zn^2+^-dependent C^+^ subtype ribosomal protein so that the ribosomal function of the cell can be maintained under Zn^2+^ limitation (Kandari et al., 2021). Furthermore, the Zn^2+^ in the C^+^ subtype ribosomal protein can also be re-released by the cell so that the ribosomal protein may be used as a large zinc reservoir (Gabriel and Helmann, 2009). RpmE and RpmJ belong to the C^+^ subtype ribosomal protein, while RpmE2 and RpmJ2 belong to the C^–^ subtype ribosomal protein. There is a Zur binding site in the promoter of the *rpmE2*-*rpmJ2* operon, and its expression is significantly upregulated under zinc starvation. The induced expression of these C^–^ subtype ribosomal proteins is an adaptive strategy of *P. aeruginosa* to zinc starvation, which functionally replaces the Zn^2+^-dependent C^+^ subtype ribosomal proteins RpmE and RpmJ (Pederick et al., 2015). This finding suggests that *P. aeruginosa* can change the distribution of intracellular Zn^2+^ to adapt to zinc starvation by converting subtype ribosomal proteins from the C^+^ to C^–^ subtype.

Another example of protein DksA2 (PA5536), a paralog protein to DksA (PA4723), expressed under zinc deficiency was observed. DksA is a Zn^2+^-dependent transcription regulatory protein in *P. aeruginosa*. During nutrient deprivation, the global regulator DksA works in conjunction with the ppGpp/pppGpp alarmone to induce extensive reprogramming of transcription and metabolism, engaging a stringent response (Gourse et al., 2018; Min and Sang, 2020). DksA coordinates Zn^2+^ through a canonical Cys4 Zn-finger motif (consisting of two Cys–X–X–Cys domains), similar to the C-site of Zur that is essential for proper folding and hence activity (Furman et al., 2013). The *P. aeruginosa* genome encodes a paralog that is closely related to DksA, called DksA2, which lacks two of the four cysteines. DksA2 contains a CxxT-(x17)-CxxA motif instead of the typical Cys4 Zn-finger domain (Blaby-Haas et al., 2011; Furman et al., 2013). Interestingly, in low-zinc environments, a *dksA2* deletion has been shown to lead to growth defects, suggesting this protein plays a role in adaptation to zinc deficiency. Indeed, under zinc starvation conditions, DksA2 is induced and can functionally substitute for the original DksA (Furman et al., 2013). Moreover, a putative Zur binding site was identified in the promoter region of *dksA2*. In addition to uptake systems, Zur could repress *dksA2* in accordance with the expression of this protein under zinc-depleted conditions (Blaby-Haas et al., 2011; Pederick et al., 2015). Although DksA2 is related to zinc in phenotype and regulation, its role in regulating intracellular zinc homeostasis in *P. aeruginosa* requires further study.

### Zinc Uptake Mediated by Low-Affinity Zinc Transporters

*Escherichia coli* can mediate zinc uptake by the non-specific low-affinity ZIP family transporter ZupT in low Zn^2+^ concentration *in vitro* and by the host *in vivo* (Wa̧tły et al., 2016). ZupT contains eight transmembrane helices. There is a His-rich domain between transmembrane domains 3 and 4 that has a wide range of metal-binding properties, including binding to Zn^2+^, Fe^2+^, Mn^2+^, or Cd^2+^, with an obvious preference for Zn^2+^. It is a low-affinity Zn^2+^ transport protein. Under the condition of abundant metal ions, this transport protein can rely on proton kinetic energy to complete the transport of metal ions to the intracellular region (Kambe, 2011). Through NCBI-BLAST homology searches and comparison, we found that there is also a ZupT homologous protein PA4467 in *P. aeruginosa* with an amino acid sequence that is 36.59% consistent with that of *E. coli* ZupT (b3040). Therefore, we speculate that PA4467 may play the role of ZupT in *P. aeruginosa* and transport Zn^2+^ into cells with low affinity depending on proton-motive force.

### Zinc Uptake Mediated by Siderophores

In addition to pseudopaline, *P. aeruginosa* may also secrete other compounds or proteins capable of chelating Zn^2+^ as zincophores involved in cellular uptake of Zn^2+^. *Pseudomonas putida* secretes pyridine-2,6-bis (thiocarboxylic acid) (PDTC), which can not only provide the function of a siderophore, but can also bind to Zn^2+^ (Leach et al., 2007). Similar to the process found in *Yersinia pestis*, the siderophore yersiniabactin binds Zn^2+^ and transports it into the cell through the intimal transporter YbtX (Bobrov et al., 2014). In *P. aeruginosa*, there are two main types of siderophores, pyochelin and pyoverdine. In addition to mediating iron acquisition, these molecules can also bind to other metal cations, including Zn^2+^ (Gonzalez et al., 2018). Because of the close relationship between Zn^2+^ and Fe^3+^ uptake, the siderophore of *P. aeruginosa* may play the role of zincophore and participate in zinc uptake.

### Zinc Uptake Mediated by T6SS

In *Yersinia pseudotuberculosis* and *Burkholderia thailandensis*, bacteria can also use type VI secretion system (T6SS) to participate in zinc uptake by secreting extracellular effector proteins that bind to Zn^2+^ (Wang et al., 2015; Si et al., 2017). Although no similar T6SS effector protein mediating cellular zinc uptake has been identified in *P. aeruginosa*, it has been reported that the T6SS effector protein can mediate cellular iron or copper uptake (Lin et al., 2017; Han et al., 2019). Our group found that the effector protein TseF is secreted by *P. aeruginosa* H3-T6SS and incorporated into outer membrane vesicles (OMVs) by directly interacting with the iron-binding PQS, a cell–cell signaling compound. The TseF-PQS-Fe^3+^ complex is then transported to the cell surface through OMVs that bind to its outer membrane receptor FptA or OprF, which mediate the entry of PQS-Fe^3+^ into cells. As a result, the uptake of extracellular Fe^3+^ by cells is completed (Lin et al., 2017). Similarly, the H2-T6SS of *P. aeruginosa* secretes a Cu^2+^-binding protein, Azu, which mediates entry of Cu^2+^ into the cell through the outer membrane receptor OprC by interacting with the OprC protein, thus accomplishing the cellular uptake of extracellular copper ions (Han et al., 2019). Therefore, *P. aeruginosa* may also participate in zinc uptake by secreting effector proteins that can bind to zincophore or Zn^2+^ through T6SS.

### Non-specific Membrane Transporters Mediate Zn^2+^ Transport

Major facilitator superfamily (MFS) proteins are a class of transport membrane proteins that move secondary metabolites in response to ion concentration gradients (Wu et al., 2020). MFS proteins have a variety of transport substrates that can carry monosaccharides, drug molecules, coenzyme factors, peptides, oligosaccharides, nucleotides, iron chelates, anions and cations, and other ligands (Saier and Paulsen, 2001; Newstead et al., 2014). Therefore, it is speculated that MFS proteins may also be involved in zinc uptake by *P. aeruginosa*.

Some studies have found that non-specific porins on the cell membrane may also be involved in the transport of Zn^2+^ from the outer membrane to the periplasmic space. *P. aeruginosa* regulates the expression of specific porins in response to Zn^2+^ availability, such as OprD (Bahr et al., 2021). In addition, OprF is the most abundant non-lipoprotein outer membrane porin in *P. aeruginosa*, allowing ions and low molecular weight sugar molecules to pass through (Chevalier et al., 2017). Therefore, porins such as OprD and OprF may also be involved in zinc uptake.

### Histidine-Mediated Uptake of Zn^2+^ Into Cells

An increasing number of studies have shown that the addition of histidine can increase the solubility of Zn^2+^ and availability of zinc transporters or that Zn^2+^ and histidine can be co-transported across membranes (Murphy et al., 2011). A study of *A. baumannii* revealed that HutUHTIG, a histidine catabolic system, participates in Zn^2+^ uptake by hydrolyzing zinc-chelating L-His (Nairn et al., 2016). In this process, the extracellular Zn^2+^ chelates with histidine to form a His-Zn^2+^ complex, which can be transported into the cell by the histidine importer HutT on the outer membrane of *A. baumannii*. ZigA is a zinc metallochaperone that shows upregulated expression in response to zinc starvation and is negatively regulated by Zur, while HutH is a Zn-Binding histidine ammonia lyase with activity that is activated by zinc. When zinc-starved, ZigA helps HutH bind to Zn^2+^, which activates HutH, and then decompresses HutT from the extracellular importation of the His-Zn^2+^ complex, releasing Zn^2+^ and producing urocanic acid. Urocanic acid is then converted into L-glutamic acid through the action of the hydrase HutU and hydrolases HutI and HutG to complete the extracellular Zn^2+^ uptake by *A. baumannii* (Nairn et al., 2016). Interestingly, HutUHTIG, a catabolic system of amino acids, also exists in *P. aeruginosa* (Capdevila et al., 2016). These results suggest that HutUHTIG may also play a role in zinc uptake in *P. aeruginosa*.

## Conclusion

During infection, hosts and pathogens compete fiercely for zinc. In this process, host cells use specifically expressed ZIP/ZnT family proteins to redistribute zinc to various tissues, which not only reduces the concentration of Zn^2+^ that pathogens can contact, but also enhances the bactericidal ability of immune cells such as macrophages and neutrophils. In addition, some zinc-sequestering proteins are expressed and recruited to the infection site to chelate zinc ions, such as calprotectin and calgranulin C, which are essential for strategic nutritional immunity. By limiting the uptake of available Zn^2+^, the host can not only effectively inhibit the growth and increment of pathogens, but also reduce the virulence and pathogenicity of pathogens.

Therefore, to ensure a successful infection, *P. aeruginosa* must adapt to the environment of zinc deficiency, and this process is mainly mediated by zinc uptake systems. Although some progress has been made in the study of zinc uptake systems of *P. aeruginosa*, many questions regarding the process of zinc uptake remain. For example: do several potential zinc ion uptake systems that have not been proven exist? If so, what are their mechanisms of action? Additionally, several known zinc uptake systems work independently; however, it is not clear how they are coordinated or if there is a distinction between primary and secondary systems. Moreover, it is not known if all of these zinc uptake systems play a role in competing with the host for zinc ions or their mechanisms of action. Only a more in-depth study of zinc uptake can provide new ideas and methods for the development of new drugs targeting *P. aeruginosa* zinc uptake systems and corresponding treatments for infection.

## Author Contributions

JL conceptualized the article and critically revised the work. SW, JC, YN, PL, and XZ performed the literature search and wrote the manuscript. SW and JC prepared the figures. All authors read and approved the final manuscript.

## Conflict of Interest

The authors declare that the research was conducted in the absence of any commercial or financial relationships that could be construed as a potential conflict of interest.

## Publisher’s Note

All claims expressed in this article are solely those of the authors and do not necessarily represent those of their affiliated organizations, or those of the publisher, the editors and the reviewers. Any product that may be evaluated in this article, or claim that may be made by its manufacturer, is not guaranteed or endorsed by the publisher.

## Footnotes

1
http://www.cbs.dtu.dk/services/SignalP/
